# Supplementary material for: Irrelevance of linear controllability to nonlinear dynamical networks
Source: Nat Commun. 2019 Sep 3;10:3961. doi: 10.1038/s41467-019-11822-5 (PMC6722065; doi:10.1038/s41467-019-11822-5)
Supplement: Supplementary file 2 — Supplementary Information [file 41467_2019_11822_MOESM2_ESM.pdf]

Supplementary Information for

Irrelevance of linear controllability to nonlinear dynamical networks

Junjie Jiang and Ying-Cheng Lai

Supplementary Note 1

We report results from a linear controllability analysis of *C. elegans* connectome, whose network structure is shown in Fig. 1(a). In a recent work [1], the neural network was treated as a linear, time invariant dynamical system with control input signals applied to sensory neurons. It was found that such a control signal would propagate to some motor neurons, and the removal of one such neuron (that had not been identified previously) would affect the muscle movement or function [1]. We have calculated that the size of the minimum controller set is quite large: $N_D = 101$, which means that, since there are only 86 sensory neurons in *C. elegans* connectome, it is not possible to control the linear network even when each and every sensory neuron receives one independent driving signal. There are many possible ways to place the required $N_D = 101$ control signals in the network, leading to many configurations of the minimum controller set. We find that a typical realization of the set contains both motor neurons and muscle cells. Figures 1(b) and 1(c) display two examples of the minimum controller set, where the driver nodes are represented by black dots. The two realizations share 43 common driver nodes, and the number of distinct drivers is 58. Note the appearance of some muscle cells in both realizations. Utilizing 1000 random realizations, we calculate the linear control importance ranking, as shown in Fig. 1(d). It can be seen that the statistical distribution of the importance is approximately uniform for most nodes in the network, with only a few exceptions. There is a probability for almost any neuron or muscle cell to belong to some specific realization of the minimum controller set. We find that the average values of the linear control importance for the three groups of neurons (sensory, inter- and motor neurons) are approximately the same: $\langle R_L \rangle_{SN} \approx 0.230$, $\langle R_L \rangle_{IN} \approx 0.211$, and $\langle R_L \rangle_{MN} \approx 0.221$. However, the average linear nodal importance for muscle cells is higher: $\langle R_L \rangle_{MC} \approx 0.399$. These data indicate that the neurons in the connectome have equal chance to be selected as a driver node, but a muscle cell is almost twice more likely to appear in the minimum controller set. This result contradicts a general understanding from both the biological and control perspectives, and has intriguing implications to the relevance of the linear controllability theory to *C. elegans* connectome. Specifically, from the point of view of biology, neurons send signals to the muscle cells, but not the other way around. From the standpoint of actual control of the network, a biologically meaningful driver set should favor neurons. Yet the linear controllability theory gives the opposite result, in contrast to the claim in Ref. [1].

In Ref. [1], some particular signal paths from the sensory neurons to a special motor neuron were identified and deemed to be particularly important based on the linear controllability theory. Does linear control really reveal any specially important motor neurons, i.e., are there any differences among the motor neurons in terms of linear control importance? To address this question, we map out all the direct paths among the sensory and motor neurons that control the muscle cells and hence the movement of *C. elegans*. Figure 2(a) shows the total numbers of direct paths of length l less than or equal to five, six, and seven from the sensory neurons to each and every motor neuron, where the abscissa is the motor neuron index. The number of these paths is large. For example, for $l \leq 7$, for each and every motor neuron, there are between 10^6 and 10^8 such paths. Apart from statistical fluctuations, the numbers of paths are approximately constant across all the

motor neurons, suggesting the nonexistence of any special motor neuron. A matrix representation of the paths for $l \leq 7$ is shown in Fig. 2(b). Between each and every pair of sensory and motor neurons, the number of such paths is at least of the order of 10^6, although the numbers associated some specific paths can be about two orders of magnitude higher.

Supplementary Note 2

We present results to corroborate Fig. 2 in the main text. Especially, Fig. 3 shows the dependence of the nonlinear and linear control importance on the betweenness centrality measure of the species. Figure 4 shows dependence of the nonlinear and linear control importance on the actual degree of the species, and Fig. 5 shows the unscaled nonlinear and linear importance of the four real mutualistic networks. All the results corroborate those in the main text in that, for the pollinators, their ranking of linear control importance exhibits a trend *opposite* to that of nonlinear control importance.

Supplementary Note 3

We study two classes of nonlinear dynamical networks: a collection of mutualistic pollinator-plant networks from the real world and an actual gene regulatory network.

Firstly, in ecology, a mutualistic network consists two groups of species, where species within the same group do not interact directly with each other - interactions or coupling occur only among the species belonging to different groups. That is, such a network necessarily possesses a bipartite structure. A representative class of such networks is the pollinator-plant networks [2, 3, 4, 5, 6, 7, 8, 9], where the pollinator species belong to one group and the plant species to another. There is no direct coupling between any two pollinator species, nor any between any pair of plant species. The interactions are constrained to being mutualistic between a pollinator species and a plant species. Mathematically, a model of pollinator-plant mutualistic network contains the following nonlinear dynamical processes: intrinsic growth, intraspecific and interspecific competitions, and the mutualistic interactions between the pollinator and plant species. Say the network has N_A pollinator species and N_P plant species. The phase space dimension of the nonlinear network is $N = N_A + N_P$, as N first-order nonlinear differential equations are needed to describe the system. Specifically, an ecologically detailed description of the pollinator-plant mutualistic network is

given by

$$\frac{dA_i}{dt} = A_i \left(\alpha_i^{(A)} - \sum_{j=1}^{N_A} \beta_{ij}^{(A)} A_j + \frac{\sum_{k=1}^{N_P} \gamma_{ik} P_k}{1 + h \sum_{k=1}^{N_P} \gamma_{ik} P_k} \right) + \mu_A, \tag{1}$$

$$\frac{dP_i}{dt} = P_i \left(\alpha_i^{(P)} - \sum_{j=1}^{N_P} \beta_{ij}^{(P)} P_j + \frac{\sum_{k=1}^{N_A} \gamma_{ik} A_k}{1 + h \sum_{k=1}^{N_A} \gamma_{ik} A_k} \right) + \mu_P, \tag{2}$$

where A_i and P_i are the abundances of the ith pollinator and the ith plant, respectively, and γ denotes the set of node-dependent parameters characterizing the strength of the mutualistic interactions in the network. For a particular mutualistic link between species i (pollinator or plant) and j (plant or pollinator), its strength can be written as

$$\gamma_{ij} = \varepsilon_{ij} \frac{\gamma_0}{(K_i)^t}, \tag{3}$$

where ϵ_{ij} defines the network structure ($\epsilon_{ij} = 1$ if there is a link between i and j; otherwise, $\epsilon_{ij} = 0$), γ_0 is the average level of mutualistic strength ($\gamma = 0$ indicates total absence of any such interaction in the network), and K_i is the number of mutualistic links of the species that benefit from the interactions. The parameter $0 \leq t \leq 1$ determines the strength of the tradeoff between the interaction strength and the number of interactions. For $t = 0$ (no tradeoff), the detailed network topology will have no effect on the mutualistic interactions. For $t = 1$ (full tradeoff), the effect of the network structure on the interactions is fully taken into account.

The physical meanings of other parameters in Equation (1) are as follows. The intraspecific and interspecific competitions are characterized by the parameters β_{ii} and $\beta_{ij}(i \neq j)$, respectively, where $\beta_{ii} \gg \beta_{ij}$. Parameter α is the intrinsic growth rate of any species in the absence of intraspecific and interspecific competitions, without any mutualistic effects. The immigration of pollinators and plants is taken into account through the respective parameters $\mu_A > 0$ and $\mu_P > 0$, which are typically weak and hardly affect the network dynamics. When the abundances of both mutualistic partners are high, the beneficial role of the mutualistic interactions in the population growth will saturate, and this effect is characterized by the half-saturation constant h.

Secondly, for a gene regulatory network of N genes, the evolution of the activity level x_i of the ith gene is governed by the following first-order nonlinear differential equation:

$$dx_i/dt = -Bx_i^f + C \sum_{j=1}^{N} A_{ij} x_j^h / (x_j^h + 1) \text{ for } i = 1, \ldots, N, \tag{4}$$

where the first term on the right-hand side models either degradation ($f = 1$) or dimerization ($f = 2$), and the sum of N Hill functions represents the genetic activation, with the Hill exponent

h and parameter $0 \leq C \leq 1$ characterizing the level of cooperation in gene regulation and the normalized maximum expression level, respectively. For $C > 0$, the network is not functional because Equation (4) permits only a trivial steady solution: $x_i = 0$ for $i = 1, \ldots, N$, which is a kind of global "extinction" state. The network is functional for $C \lesssim 1$. It is thus convenient to use C as a natural bifurcation parameter of the nonlinear network system.

Supplementary Tables

(1) Anastoechus, latifrons	(2) Anthophora, alluaudi	(3) Apis, mellifera	(4) Euodynerus, reflexus
(5) Geron, hesperidon	(6) Eristalis, tenax	(7) Megachile, canariensis	(8) Anthrax, anthrax
(9) Eucera, gracilipes	(10) Hyleaus, canariensis	(11) Lasioglossum, viride	(12) Linnaemyia, soror
(13) Cephalodromia	(14) Cyclyrius, webbianus	(15) Estheria, simonyi	(16) Lasioglossum, actifrons
(17) Melecta, curvispina	(18) Osmia, canariensis	19) Andrena, wollestoni	(20) Colletes, dimidiatus
(21) Gasteruption	(22) Lucilia, sericata	(23) Macroglossum, stellatarum	(24) Scaeva, albomaculata
(25) Stomorhina, lunata	(26) Unidentified	(27) Anthidium, manicatum	(28) Bibio, elmoi
(29) Dermasothes, gracile	(30) Drosophila	(31) Lasioglossum, chalcodes	(32) Leptochilus, eatoni
(33) Nyctia, lugubris	(34) Peleteria, ruficornis	(35) Phylloscopus, collybita	(36) Serinus, canarius
(37) Tachina, canariensis	(38) Tachysphex, unicolor		

Supplementary Table 1. All names of the species in Fig. 1 in the main text.

Index	# Pollinators	# Plants	Linkage	Network Location
1	101	84	0.04	Cordn del Cepo, Chile
2	64	43	0.07	Cordn del Cepo, Chile
3	25	36	0.09	Cordn del Cepo, Chile
4	102	12	0.14	Central New Brunswick, Canada
5	275	96	0.03	Pikes Peak, Colorado, USA
6	61	17	0.14	Hickling, Norfolk, UK
7	36	16	0.15	Shelfanger, Norfolk, UK
8	38	11	0.25	Tenerife, Canary Islands
9	118	24	0.09	Latnjajaure, Abisko, Sweden
10	76	31	0.19	Zackenberg
11	13	14	0.29	Mauritius Island
12	55	29	0.09	Garajonay, Gomera, Spain
13	56	9	0.2	KwaZulu-Natal region, South Africa
14	81	29	0.08	Hazen Camp, Ellesmere Island, Canada
15	666	131	0.03	Daphn, Athens, Greece

Supplementary Table 2 – continued from previous page

Index	# Pollinators	# Plants	Linkage	Network Location
16	179	26	0.09	Doana National Park, Spain
17	79	25	0.15	Bristol, England
18	108	36	0.09	Hestehaven, Denmark
19	85	40	0.08	Snowy Mountains, Australia
20	91	20	0.1	Hazen Camp, Ellesmere Island, Canada
21	677	91	0.02	Ashu, Kyoto, Japan
22	45	21	0.09	Laguna Diamante, Mendoza, Argentina
23	72	23	0.08	Rio Blanco, Mendoza, Argentina
24	18	11	0.19	Melville Island, Canada
25	44	13	0.25	North Carolina, USA
26	54	105	0.04	Galapagos
27	60	18	0.11	Arthur's Pass, New Zealand
28	139	41	0.07	Cass, New Zealand
29	118	49	0.06	Craigieburn, New Zealand
30	53	28	0.07	Guarico State, Venezuela
31	49	48	0.07	Canaima Nat. Park, Venezuela
32	33	7	0.28	Brownfield, Illinois, USA
33	34	13	0.32	Ottawa, Canada
34	128	26	0.09	Chiloe, Chile
35	36	61	0.08	Morant Point, Jamaica
36	12	10	0.25	Flores, Aores Island
37	40	10	0.18	Hestehaven, Denmark
38	42	8	0.24	Hestehaven, Denmark
39	51	17	0.15	Tenerife, Canary Islands
40	43	29	0.09	Windsor, The Cockpit Country, Jamaica
41	43	31	0.11	Syndicate, Dominica
42	6	12	0.35	Puerto Villamil, Isabela Island, Galapagos
43	82	28	0.11	Hestehaven, Denmark
44	609	110	0.02	Amami-Ohsima Island, Japan
45	26	17	0.14	Uummannaq Island, Greenland

Supplementary Table 2 – continued from previous page

Index	# Pollinators	# Plants	Linkage	Network Location
46	44	16	0.39	Denmark
47	186	19	0.12	Isenbjerg
48	236	30	0.09	Denmark
49	225	37	0.07	Denmark
50	35	14	0.18	Tenerife, Canary Islands
51	90	14	0.13	Nahuel Huapi National Park, Argentina
52	39	15	0.16	Tundra, Greenladn
53	294	99	0.02	Mt. Yufu, Japan
54	318	113	0.02	Kyoto City, Japan
55	195	64	0.03	Nakaikemi marsh, Fukui Prefecture, Japan
56	365	91	0.03	Mt. Kushigata, Yamanashi Pref., Japan
57	883	114	0.02	Kibune, Kyoto, Japan
58	81	32	0.12	Parc Natural del Cap de Creus
59	13	13	0.42	Parque Nacional do Catimbau

Supplementary Table 2. The 59 real pollinator-plant networks are from web-of-life (http://www.web-of-life.es). For each network, the linkage is normalized with respect to the corresponding fully connected (all-to-all) network for which the linkage is 100%.

Supplementary figures

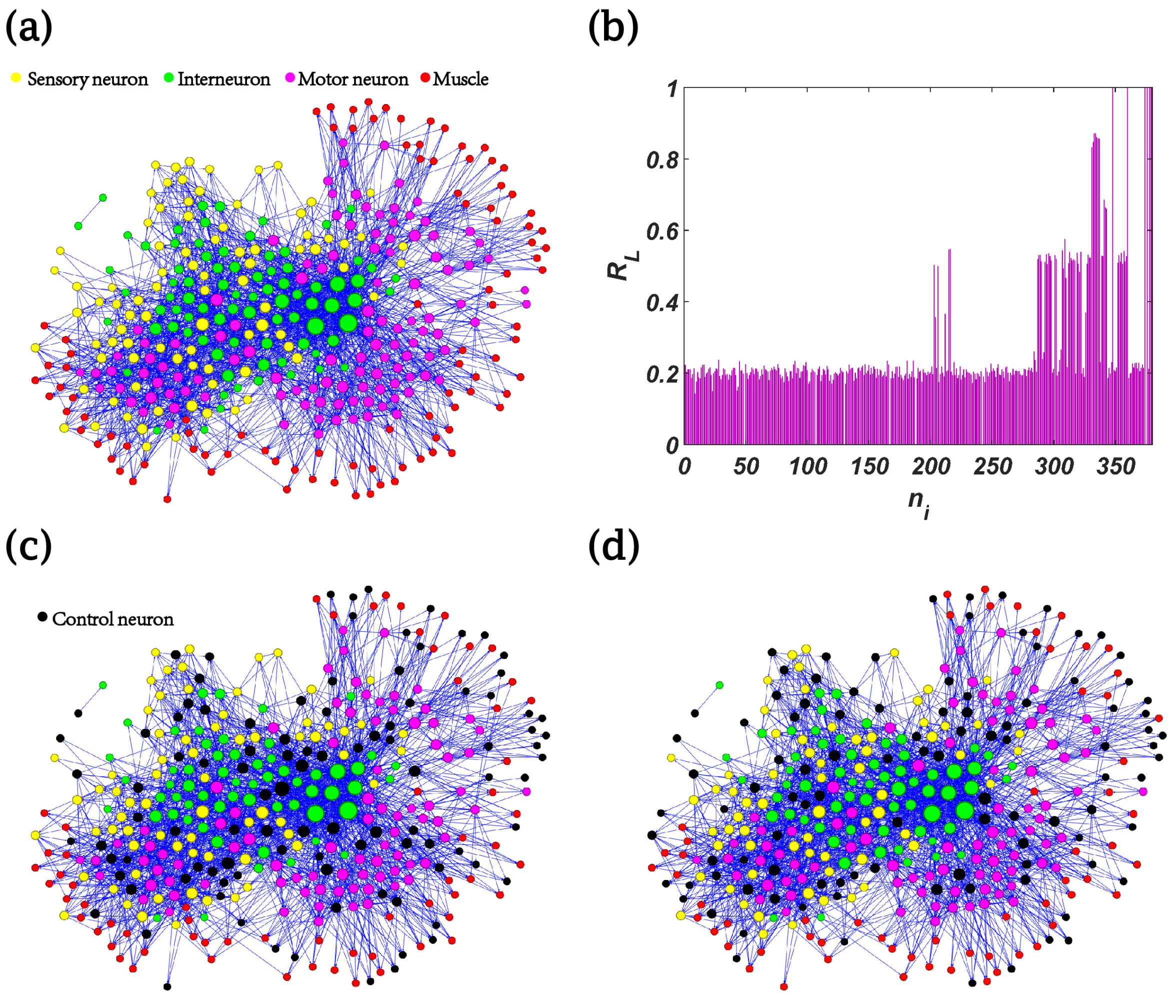

Supplementary Figure 1. Linear control importance ranking for *Caenorhabditis elegans* connectome. (a) A graphical representation of *C. elegans* connectome. The network contains 282 neurons and 97 muscle cells, where the yellow, green, magenta, and red nodes represent sensory neurons, inter-neurons, motor neurons, and muscle cells, respectively. The size of a node is proportional to the sum of its in- and out-degrees. The dynamical network is nonlinear, but a mathematical description of reasonable detail is not available at the present. When the network is artificially treated as a linear, time-invariant system, the size of the minimum controller set is $N_D = 101$. (b) Linear control importance ranking, where the index on the abscissa is arranged in a descending order of the nodal degree (sum of in- and out-degrees). The importance distribution is approximately uniform across the nodes, except for a small fraction of nodes. (c,d) Two realizations of the minimum controller set (black dots). Because of the relatively large size of the network, the number of distinct minimum controller sets is quite large.

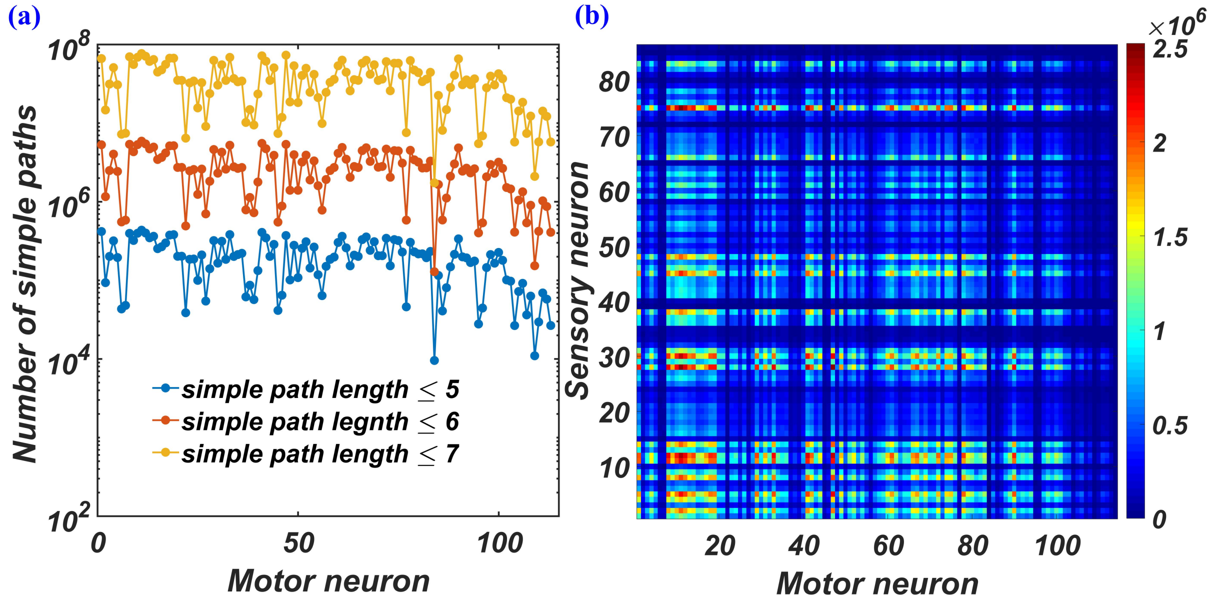

Supplementary Figure 2. Signal paths between sensory neurons and motor neurons in *C. elegans*. (a) The numbers of direct paths from all sensory neurons to motor neurons with path length less than or equal to seven (yellow), six (red), and five (blue). The numbers of such paths are enormous. (b) Matrix representation of all direct paths with path length less than or equal to seven, from each and every sensory neuron to each and every motor neuron.

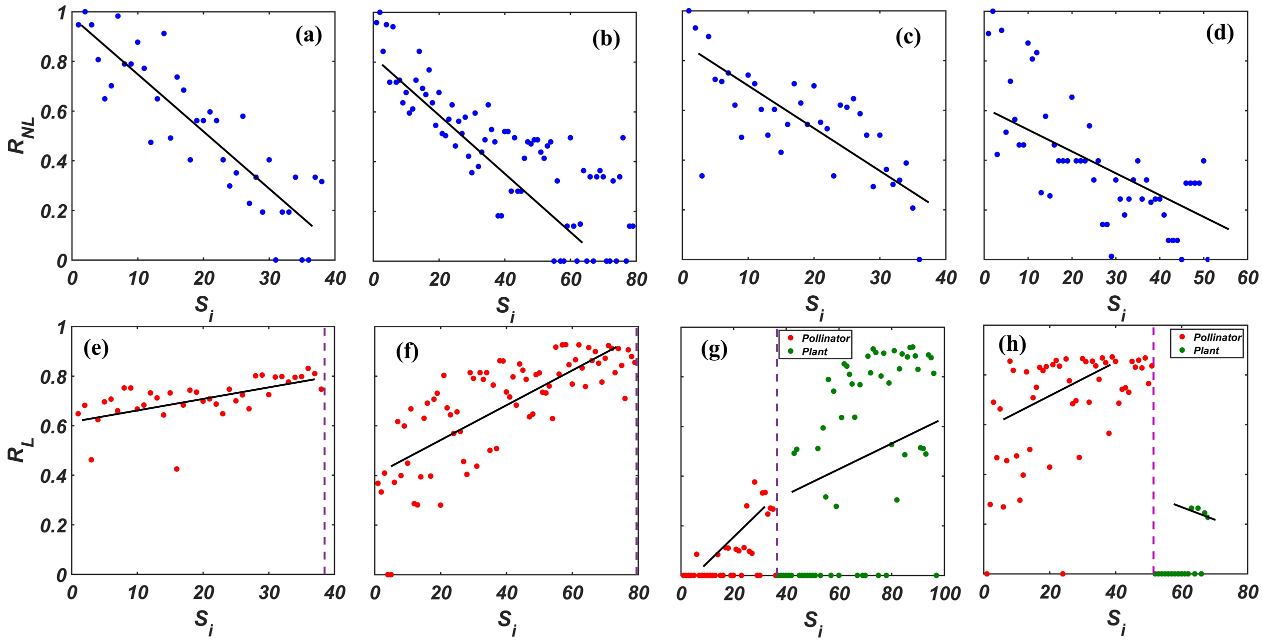

Supplementary Figure 3. Nodal importance ranking in nonlinear and linear control of mutualistic networks in terms of betweenness centrality. The four empirical networks are labeled as A, B, C, and D with details given in **Methods** in the main text. (a-d) Nonlinear and (e-h) linear control importance ranking for networks $A - D$, respectively. For tipping point control of the nonlinear network in (a-d), only the pollinator species are subject to external intervention through the managed maintenance of the abundance of a single species. The nodal index on the abscissa of each panel is arranged according to the betweenness centrality of the node: from high to low degree values (left to right). Other setting and parameter values are the same as the Fig. 2 in the main text. The betweenness centrality based results are essentially the same as those based on the degree centrality in the main text in that, for the pollinators, their ranking of linear control importance exhibits a trend *opposite* to that of nonlinear control importance.

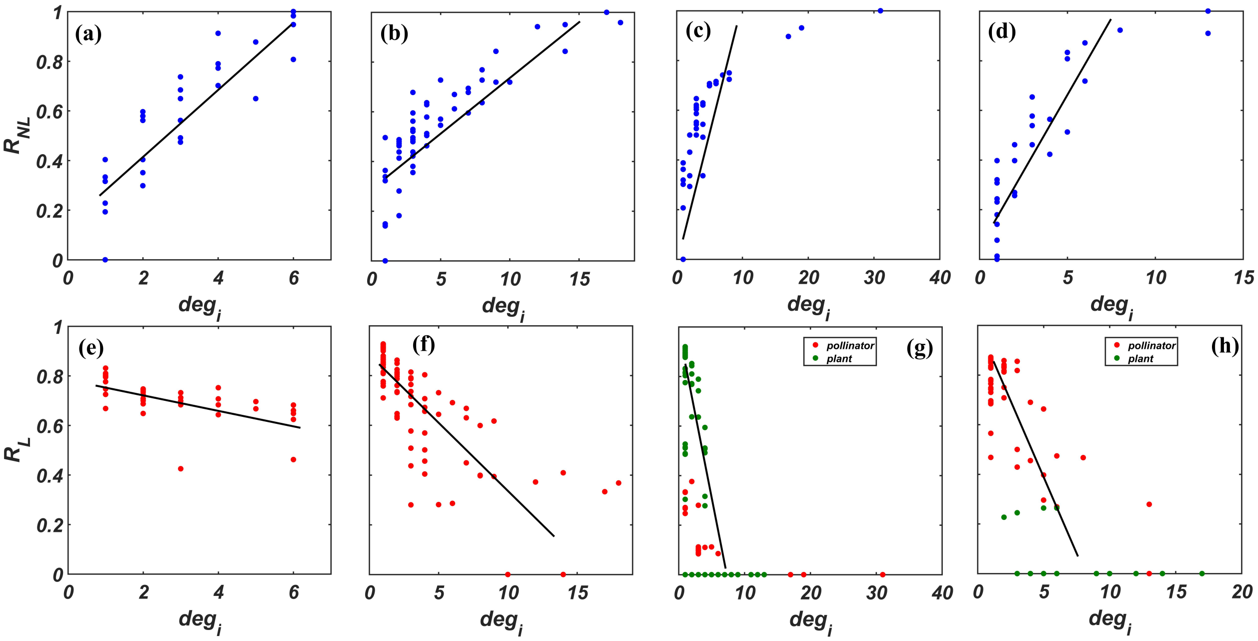

Supplementary Figure 4. Nodal importance ranking of nonlinear and linear control of mutualistic networks in terms of the actual degree values. For the four empirical networks $A - D$, (a-d) nonlinear and (e-h) linear control importance ranking, respectively. For tipping point control of the nonlinear network in (a-d), only the pollinator species are subject to external intervention through the managed maintenance of the abundance of a single species. The horizontal axis is the actual degree of the species. Other setting and parameter values are the same as Fig. 2 in the main text. The results indicate that, for the pollinators, their nonlinear and linear control importance ranking exhibits *opposite* trends, as in Fig. 2 in the main text.

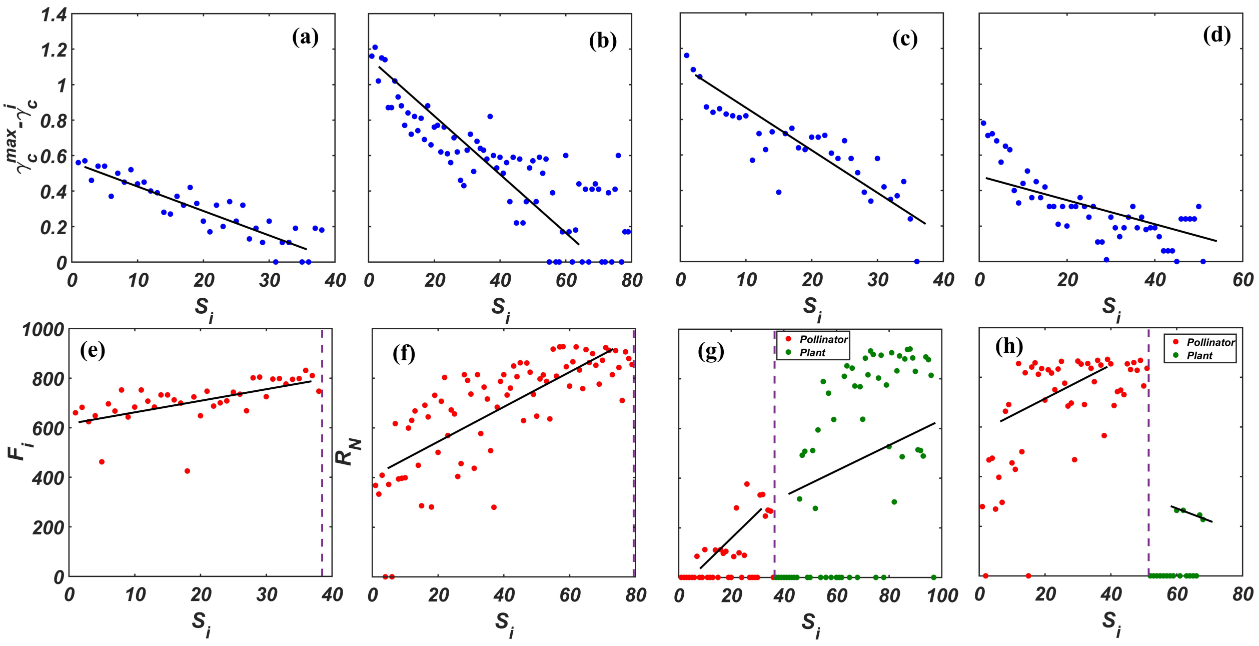

Supplementary Figure 5. Opposite behaviors of unscaled nodal importance ranking in nonlinear and linear control of pollinator-plant mutualistic networks. For the four empirical networks $A-D$, (a-d) unscaled nonlinear and (e-h) unscaled linear control importance ranking, respectively. The unscaled nonlinear control importance for species i is given by $\gamma_c^{max} - \gamma_c^i$ based on Eq. (5) in the main text. The unscaled linear control importance for species i is F_i, which is the times that the ith species appears in F number of configurations of the minimum controller sets. All other setting and parameter values are the same as Fig. 2 in the main text. For the pollinators, their ranking of linear control importance exhibits a trend *opposite* to that of nonlinear control importance.

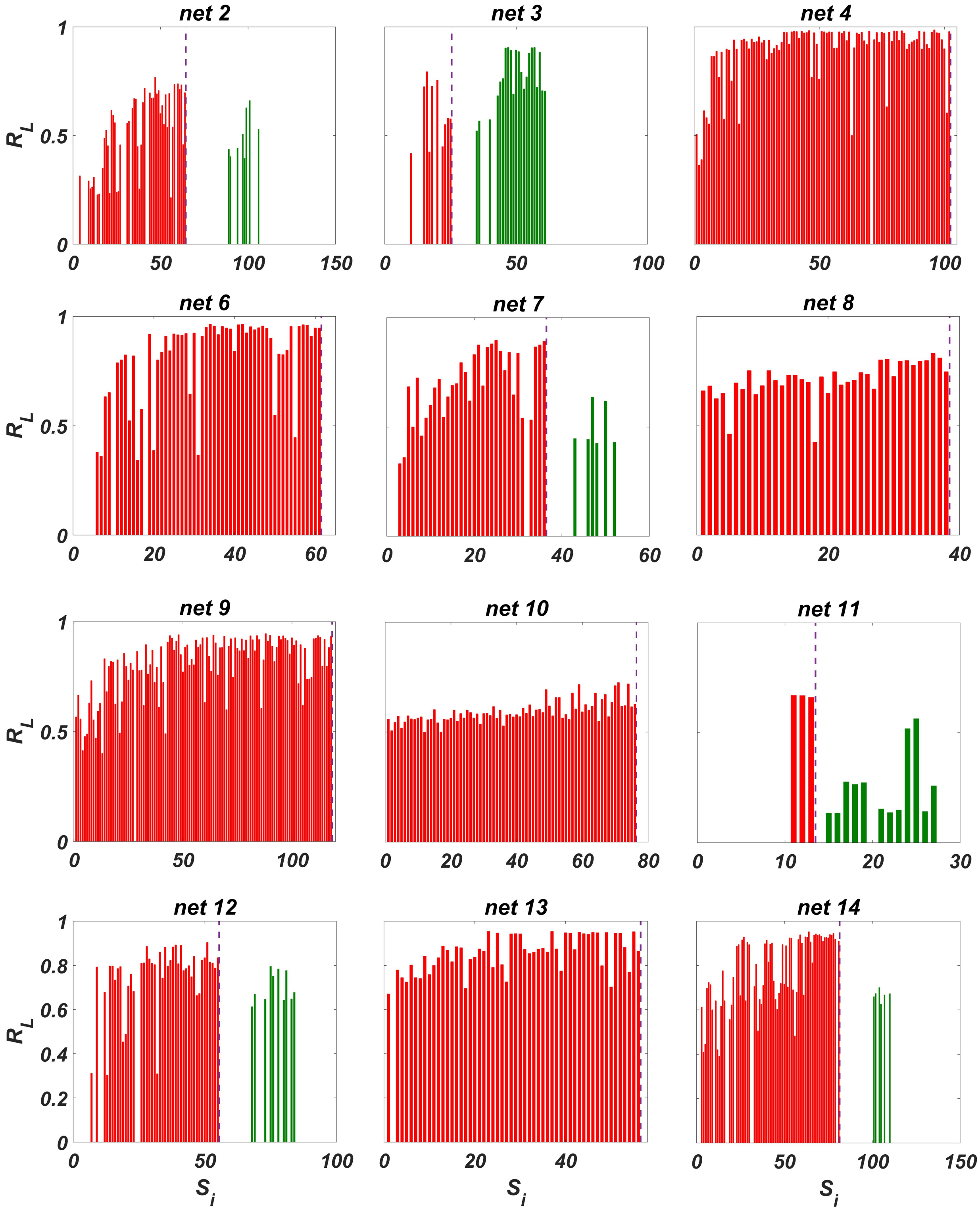

Supplementary Figure 6. Linear control importance ranking of 12 empirical pollinator-plant mutualistic networks.

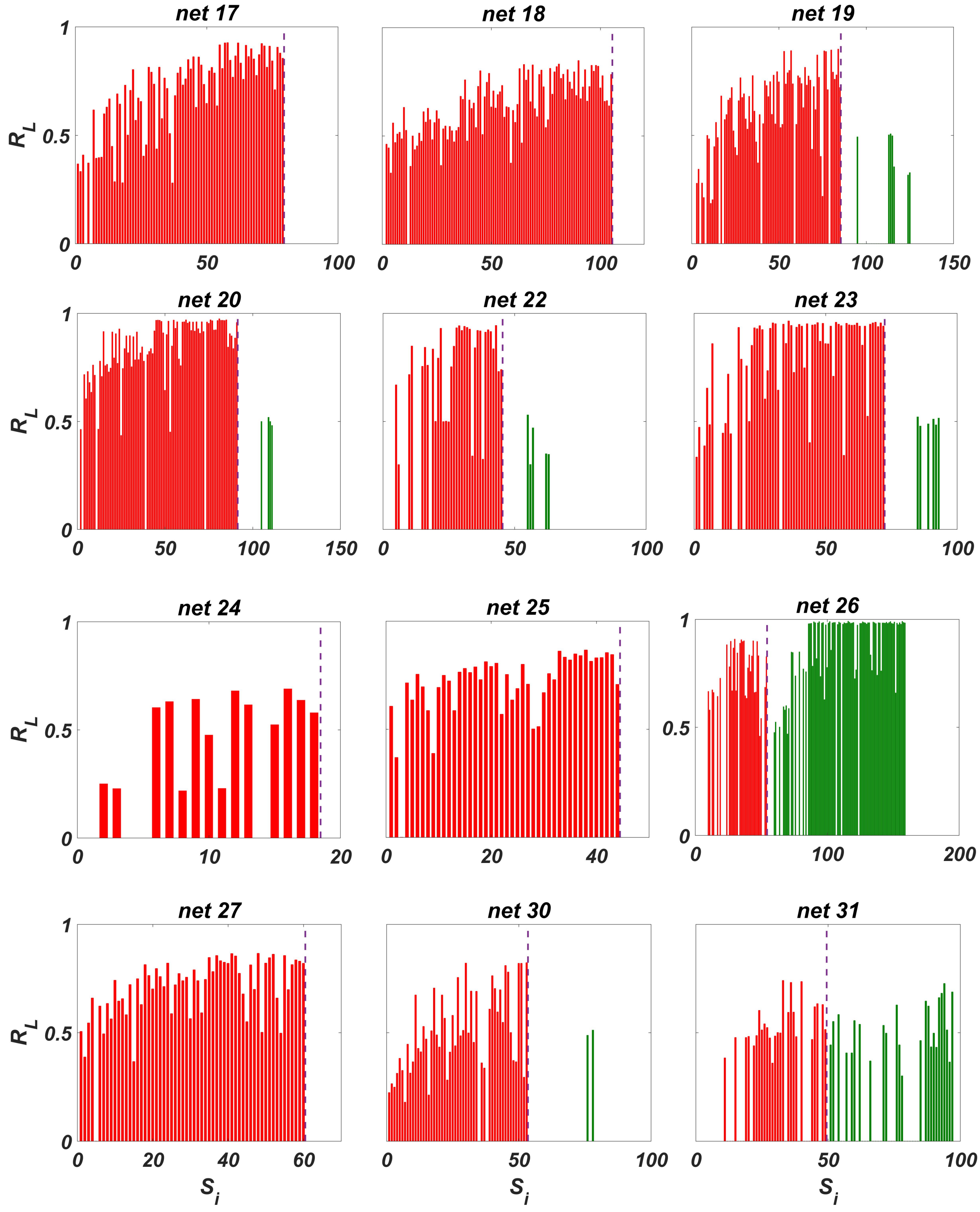

Supplementary Figure 7. **Linear control importance ranking of 12 empirical pollinator-plant mutualistic networks**.

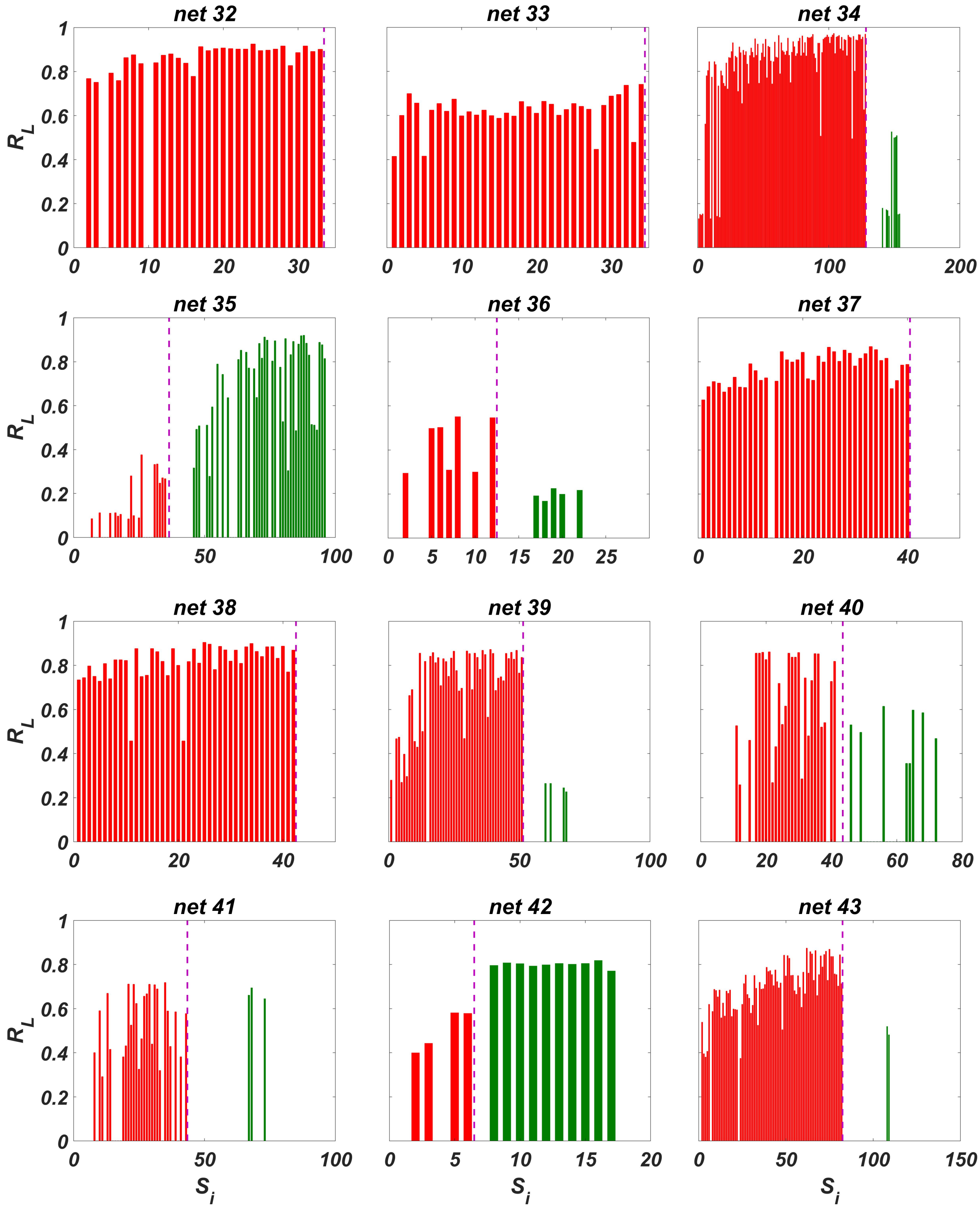

Supplementary Figure 8. Linear control importance ranking of 12 empirical pollinator-plant mutualistic networks.

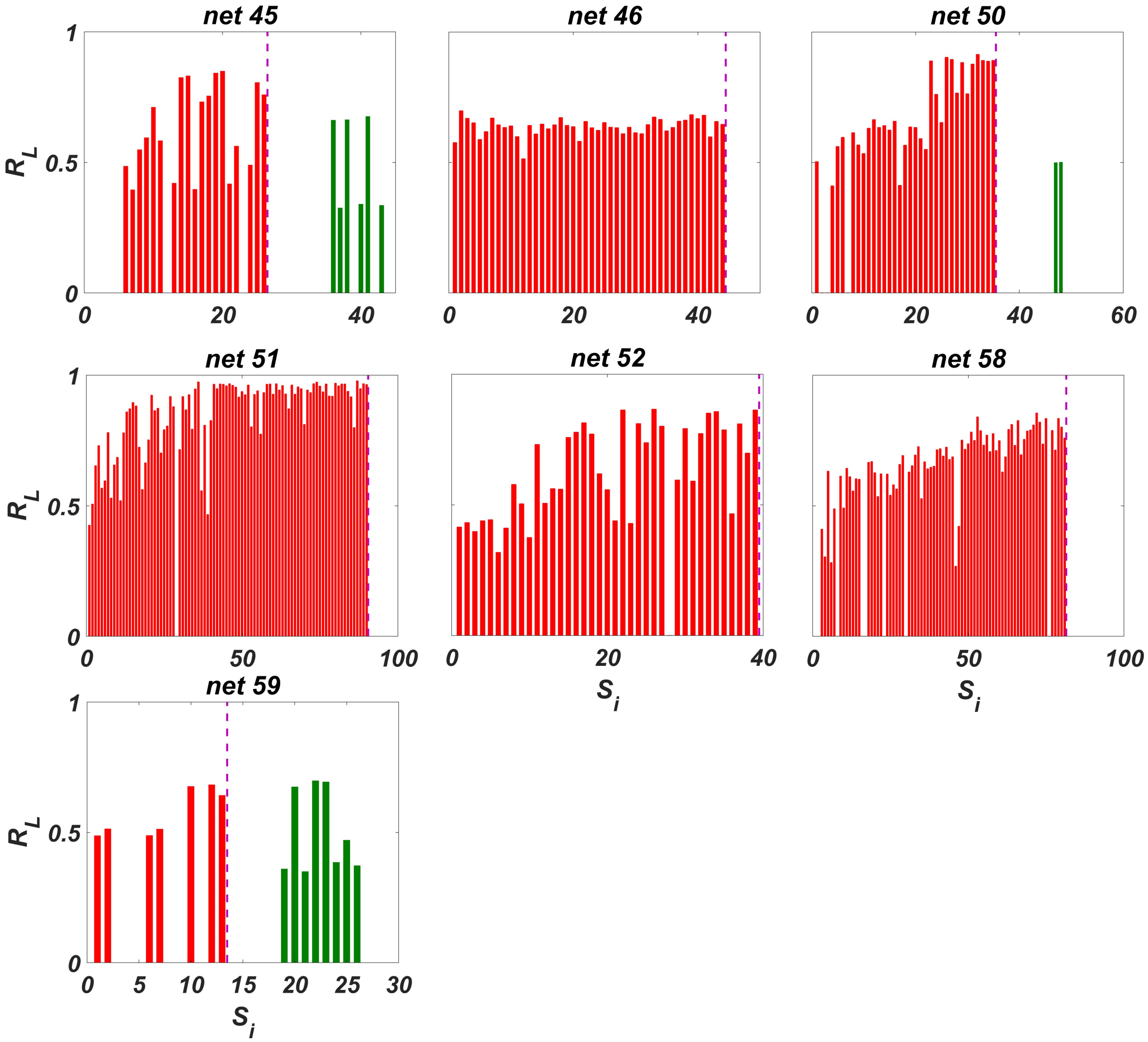

Supplementary Figure 9. Linear control importance ranking of seven empirical pollinator-plant mutualistic networks.

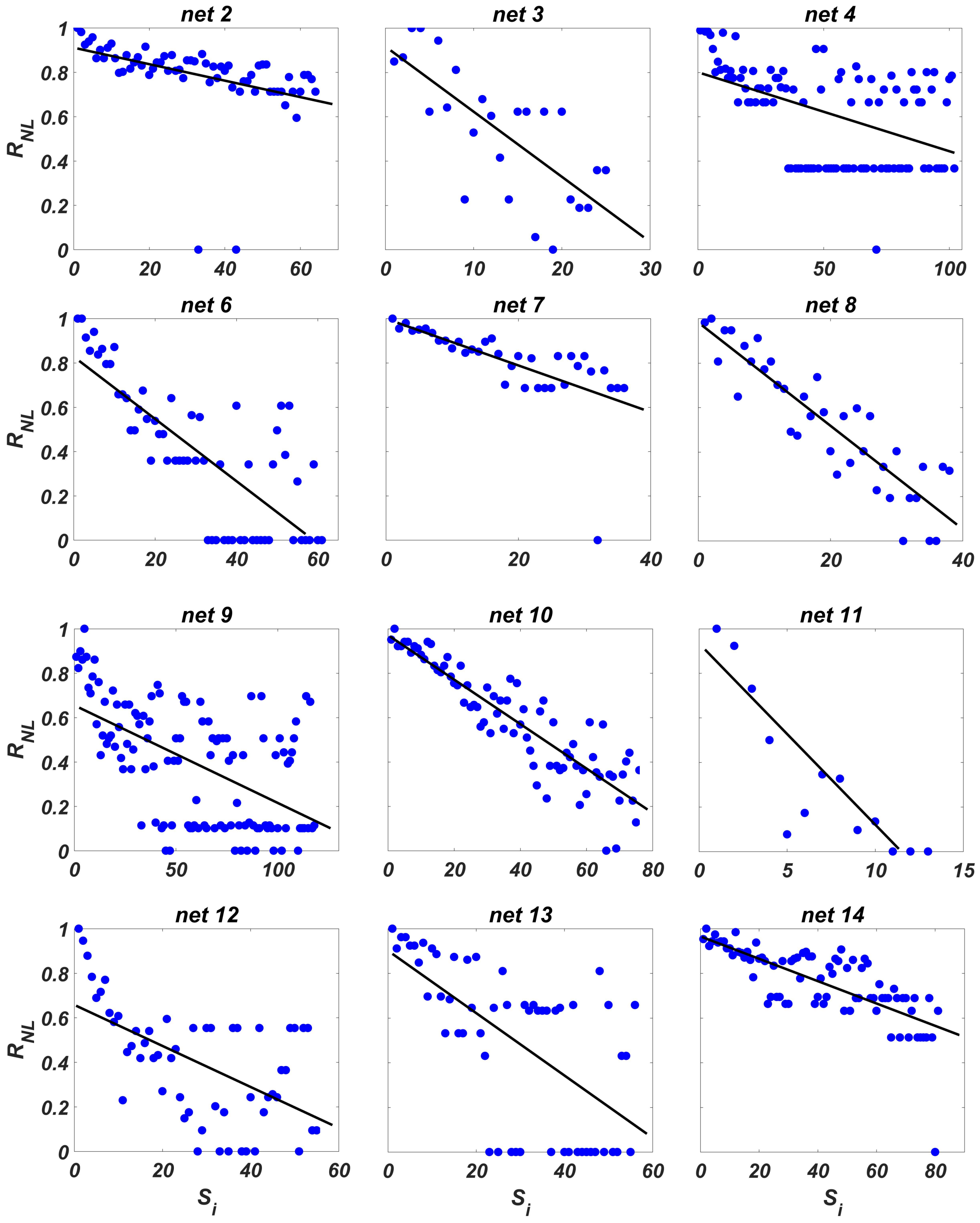

Supplementary Figure 10. Nonlinear control importance ranking of 12 empirical pollinator-plant mutualistic networks. The parameter values for the nonlinear dynamical networked system are $h = 0.2$, $t = 0.5$, $\beta_{ii}^{(A)} = \beta_{ii}^{(P)} = 1$, $\beta_{ij}^{(A)} = \beta_{ij}^{(P)} = 0$, $\alpha_i^{(A)} = \alpha_i^{(P)} = -0.3$, and $\mu_A = \mu_P = 0.0001$. The step of changes in γ_0 0.01. The controlled abundance level $A_S = 1.5$. Species with $R_N = 0$ indicates that controlling any one of them is unable to restore the populations in the aftermath of a tipping point transition.

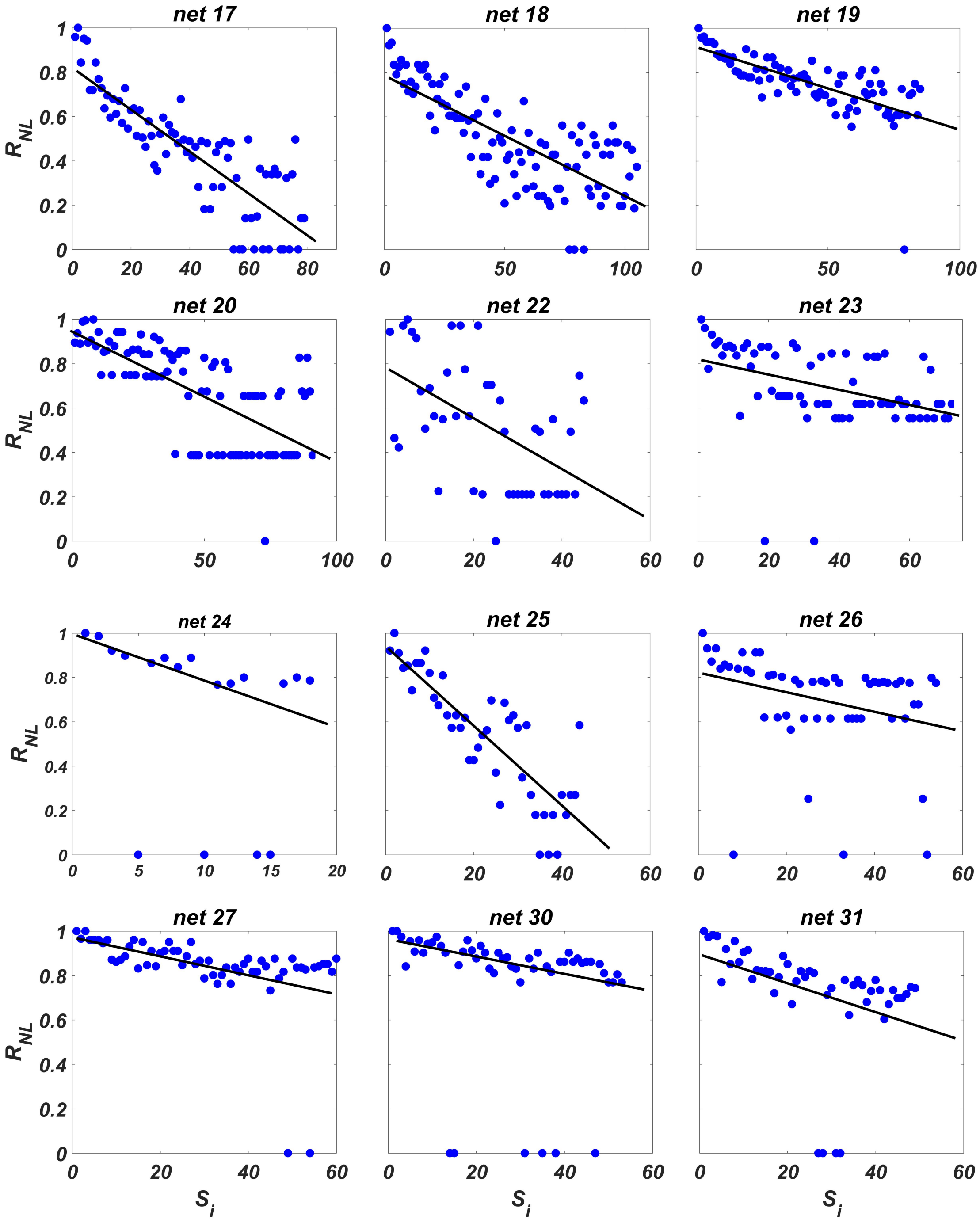

Supplementary Figure 11. Nonlinear control importance ranking of 12 empirical pollinator-plant mutualistic networks. Legends and parameter values are the same as those in Fig. 10.

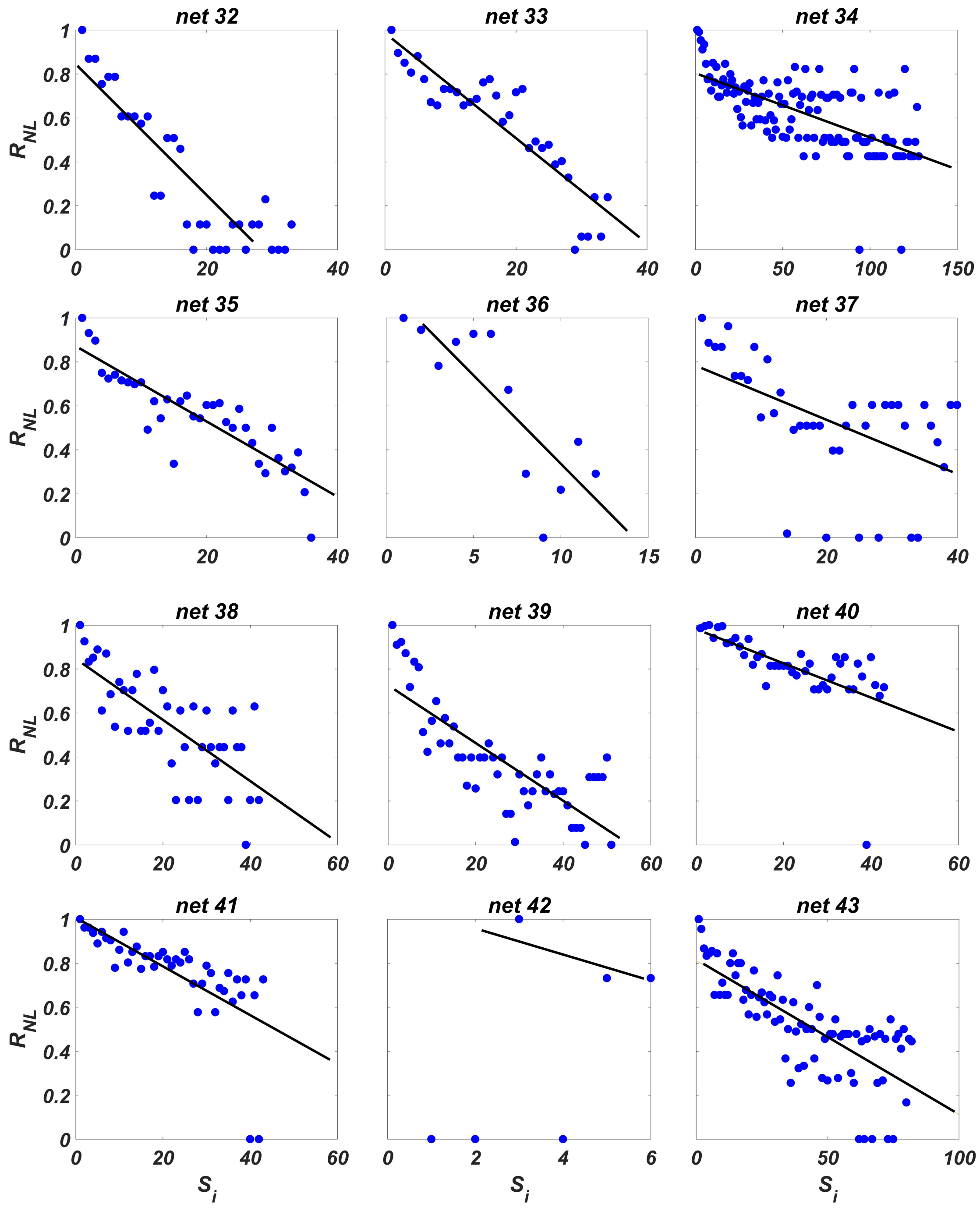

Supplementary Figure 12. Nonlinear control importance ranking of 12 empirical pollinator-plant mutualistic networks. Legends and parameter values are the same as those in Fig. 10.

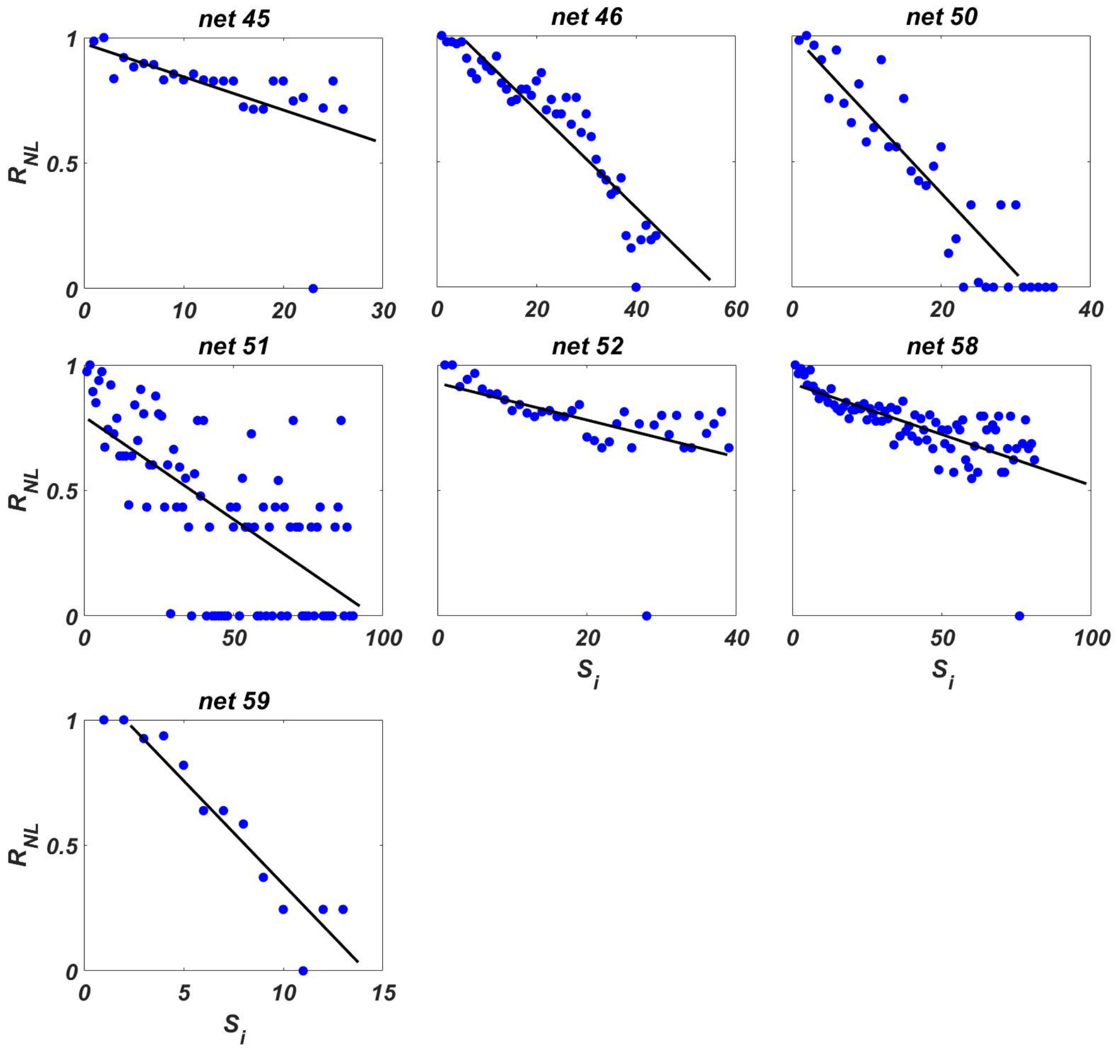

Supplementary Figure 13. Nonlinear control importance ranking of seven empirical pollinator-plant mutualistic networks. Legends and parameter values are the same as those in Fig. 10.

Supplementary References

[1] Yan, G. *et al.* Network control principles predict neuron function in the caenorhabditis elegans connectome. *Nature* **550**, 519 (2017).

[2] Bascompte, J., Jordano, P., Melián, C. J. & Olesen, J. M. The nested assembly of plant-animal mutualistic networks. *Proc. Natl. Acad. Sci. (USA)* **100**, 9383–9387 (2003).

[3] Guimaraes, P. R., Jordano, P. & Thompson, J. N. Evolution and coevolution in mutualistic networks. *Ecol. Lett.* **14**, 877–885 (2011).

[4] Nuismer, S. L., Jordano, P. & Bascompte, J. Coevolution and the architecture of mutualistic networks. *Evolution* **67**, 338–354 (2013).

[5] Lever, J. J., Nes, E. H., Scheffer, M. & Bascompte, J. The sudden collapse of pollinator communities. *Ecol. Lett.* **17**, 350–359 (2014).

[6] Rohr, R. P., Saavedra, S. & Bascompte, J. On the structural stability of mutualistic systems. *Science* **345**, 1253497 (2014).

[7] Dakos, V. & Bascompte, J. Critical slowing down as early warning for the onset of collapse in mutualistic communities. *Proc. Natl. Acad. Sci. (USA)* **111**, 17546–17551 (2014).

[8] Guimaraes, P. R., Pires, M. M., Jordano, P., Bascompte, J. & Thompson, J. N. Indirect effects drive coevolution in mutualistic networks. *Nature* **550**, 511–514 (2017).

[9] Jiang, J. *et al.* Predicting tipping points in mutualistic networks through dimension reduction. *Proc. Natl. Acad. Sci. (USA)* 201714958 (2018).
